# Mitochondrial Disease Diagnosed Following Preterm Birth at 29 Weeks of Gestation and Postpartum Heart Failure: A Case Report and Literature Review

**DOI:** 10.1002/ccr3.71532

**Published:** 2025-11-28

**Authors:** Tomoyuki Watanabe, Gen Ishikawa, Tsutomu Tabata

**Affiliations:** ^1^ Department of Obstetrics and Gynecology Tokyo Women's Medical University Tokyo Japan

**Keywords:** diabetes mellitus, mitochondrial diseases, mitochondrial DNA, pregnancy, premature birth

## Abstract

In some cases, mitochondrial disease can remain undiagnosed until pregnancy reveals systemic symptoms. Clinicians should therefore consider this diagnosis in young patients presenting with diabetes, kidney disease, and hearing loss. Early diagnosis can improve maternal and fetal outcomes, particularly in high‐risk pregnancies complicated by unexplained preterm birth or cardiomyopathy.

## 1 Introduction

Mitochondrial diseases, caused by impaired mitochondrial energy production, are characterized by a wide range of clinical symptoms primarily affecting organs with high energy demands and are the result of mutations in the maternally inherited mitochondrial deoxyribonucleic acid (mtDNA) or one of many nuclear DNA (nDNA) genes [1]. Mitochondrial dysfunction occurs when the proportion of pathogenic mtDNA variants exceeds a certain threshold; however, the severity of the disease does not always correlate with the proportion of mutant DNA in individual cells, leading to considerable variability in the clinical severity among patients, even among siblings [2], suggesting the possibility of undiagnosed mild cases. As the increased energy demands associated with pregnancy may unmask latent symptoms, careful attention is warranted. We report herein a case of mitochondrial dysfunction diagnosed after a preterm delivery (29 weeks of gestation) and subsequent postpartum heart failure.

## 2 Case History/Examination

A 37‐year‐old woman, gravida 4, para 1, presented with a history of type 2 diabetes mellitus, diagnosed at 29 years of age, and chronic tubulointerstitial nephritis, diagnosed at 35 years of age, corresponding to chronic kidney disease (CKD) stage 3. Due to her history of early‐onset diabetes, the possibility of maturity‐onset diabetes of the young (MODY) was considered; however, her insulin secretion was adequate, and insulin resistance was consistent with type 2 diabetes mellitus. Tests for glutamic acid decarboxylase antibodies and islet antigen‐2 antibodies were negative. The patient's pre‐pregnancy insulin requirement was 42 units/day, and her family history included type 2 diabetes mellitus in both maternal grandparents as well as ovarian cancer in her mother. Her two sisters had no significant medical histories. Screening for hereditary diseases was not performed in this case. The results of blood and urine tests conducted at 32 years of age are presented in Table 1.

**TABLE 1:** Laboratory data at 32 years of age.

Hematologic values			Blood chemical values			Urinalysis	
White blood cell	8,700/μL	H	AST	20 U/L		Specific gravity	1.011
Red blood cell	4.98 × 10^6^/μL		ALT	19 U/L		pH	6
Hemoglobin	14.8 g/dL		LDH	1,679 U/L		Protein	2+
Hematocrit	42.30%		Creatinine	0.66 mg/dL		Glucose	−
Platelets	24.0 × 10^4^/μL		BUN	18.3 mg/dL		Occult blood	−
			eGFR	85.9 mL/min/1.73m^2^		Urobilinogen	±
			Uric acid	6.0 mg/dL			
			Na	138 mEq/L		Urinary protein	0.124 g/dL
			K	4.4 mEq/L		Urinary creatinine	42.28 mg/dL
			Cl	102 mEq/L		Urinary protein/creatinine	2.93 g/g/Cr
			Anti‐GAD antibody	5.0 U/mL	H		
			C‐peptide	2.9 ng/mL	H		
			Hemoglobin A1c	10.30%	H		

Abbreviations: ALT, alanine aminotransferase; AST, aspartate aminotransferase; BUN, blood urea nitrogen; eGFR, estimated Glomerular filtration rate; GAD, glutamic acid decarboxylase; LDH; lactate dehydrogenase.

At 33 years of age, the patient had a spontaneous vaginal delivery at 37 weeks after hospitalization for threatened preterm labor. Magnesium sulfate was administered, but discontinued due to respiratory distress. Additionally, the patient experienced placenta accreta, which required manual removal. At 35 years of age, she experienced preterm premature rupture of membranes (pPROM) at 19 weeks, leading to a spontaneous abortion at 21 weeks. In that instance, the patient's placenta failed to detach spontaneously and required manual removal with forceps. A subsequent first‐trimester miscarriage occurred at 36 years of age.

Despite being medically advised against getting pregnant due to her comorbidities and prior pregnancy complications, the patient spontaneously conceived. She was referred to our institution for high‐risk pregnancy management at 15 weeks of gestation.

**FIGURE 1:**
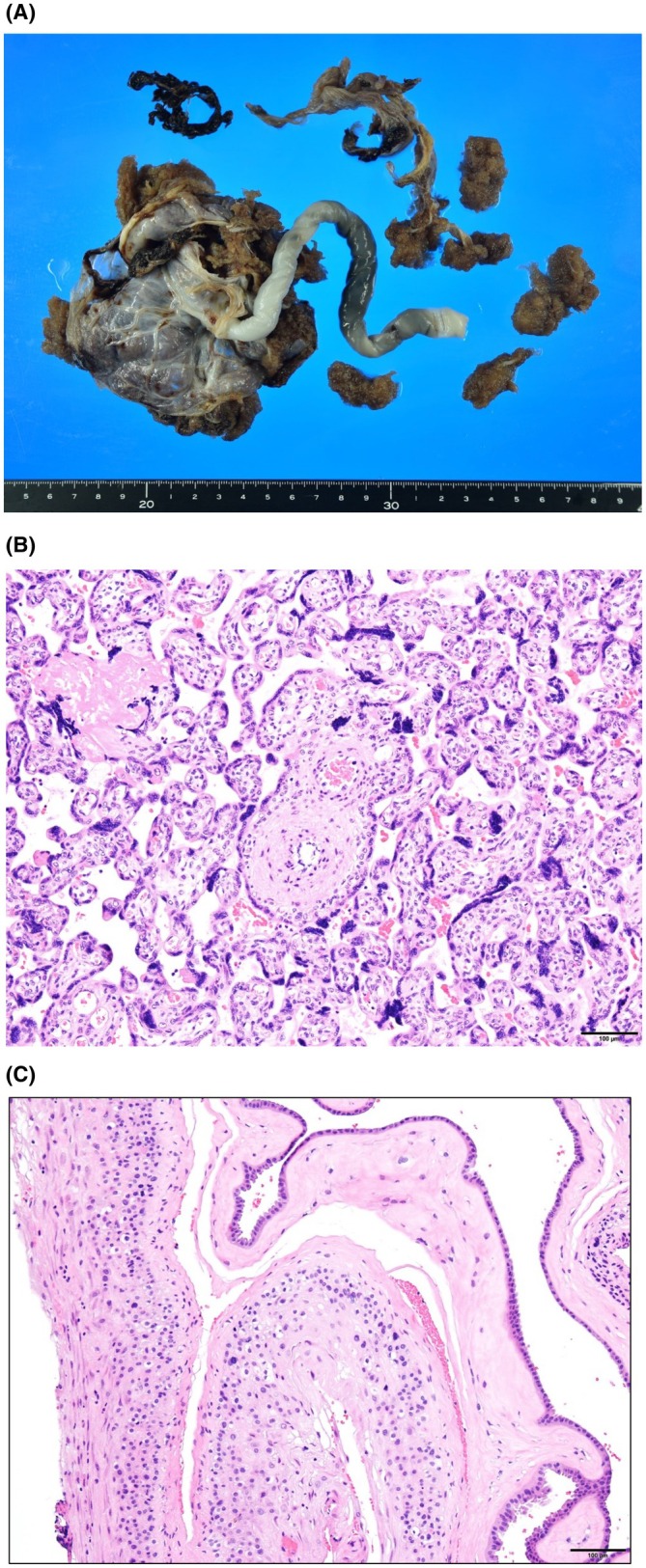
Pathological findings of the placenta. Macroscopic examination: The placenta ruptured during delivery, and a portion was retained in the uterus. Microscopic examination of the villi: An increase in syncytial knot can be observed. There is no presence of uterine smooth muscle tissue, and no evidence of placenta accreta. Microscopic examination of fetal membranes: No evidence of inflammation could be observed.

Transvaginal ultrasonography at 24 weeks of gestation demonstrated cervical shortening (24 mm). At admission, the patient's vital signs were as follows: Body temperature, 36.6°C; blood pressure, 122/68 mmHg; and pulse rate, 70 bpm. The patient reported irregular uterine contractions, leading to a diagnosis of threatened preterm labor. The patient was subsequently admitted for inpatient management and observation. Given the presence of uterine contractions, cervical cerclage was deemed inappropriate. In addition, due to prior adverse effects of magnesium sulfate and concerns regarding hyperglycemia associated with ritodrine hydrochloride, tocolytic agents were not administered at this time. Instead, the patient was managed with bed rest and close observation alone. The results of blood and urine tests at the time of admission are shown in Table 2. At 28 weeks 3 days, cervical dilation progressed with visibly bulging fetal membranes, and intravenous ritodrine hydrochloride was initiated for tocolysis; however, at 29 weeks 4 days, spontaneous preterm labor occurred, resulting in the vaginal delivery of a female infant weighing 1,046 g with Apgar scores of 3 and 7 at 1 and 5 min, respectively. The patient's placenta failed to detach spontaneously, necessitating manual extraction under general anesthesia. The placenta had adhered to the uterine wall, making detachment challenging; therefore, a portion of the placenta remained in situ. However, the histopathological analysis did not confirm a diagnosis of placenta accreta (Figure 1). Total blood loss during delivery was 1,500 mL, necessitating the transfusion of 400 mL of red blood cells.

**TABLE 2:** Laboratory data on admission (24 weeks and 4 weeks of gestation).

Hematologic values			Blood chemical values			Urinalysis	
White blood cell	14,440/μL	H	C reactive protein	0.30 mg/dL	H	Specific gravity	1.011
Red blood cell	2.81 × 10^6^/μL	L	AST	13 U/L		pH	5.5
Hemoglobin	8.4 g/dL	L	ALT	6 U/L		Protein	3+
Hematocrit	25.50%	L	LDH	160 U/L		Glucose	−
Platelets	34.2 × 10^4^/μL		Creatinine	1.74 mg/dL	H	Occult blood	−
			BUN	42.6 mg/dL	H	Urobilinogen	±
Coagulation tests			eGFR	27.7 mL/min/1.73 m^2^			
PT‐INR	0.91		Uric acid	9.6 mg/dL	H	Urinary protein	0.275 g/dL
APTT	22.3	L	Na	138 mEq/L		Urinary creatinine	37 mg/dL
AT‐III	111		K	4.8 mEq/L		Urinary protein/creatinine	7.43 g/g/Cr
Fibrinogen	526	H	Cl	107 mEq/L			

Abbreviations: ALT, alanine aminotransferase; APTT, activated partial thromboplastin time; AST, aspartate aminotransferase; AT‐III, antithrombin‐III; BUN, blood urea nitrogen; CRP, C‐reactive protein; eGFR, estimated Glomerular filtration rate; LDH, lactate dehydrogenase; PT‐INR, prothrombin time‐international normalized ratio.

**FIGURE 2:**
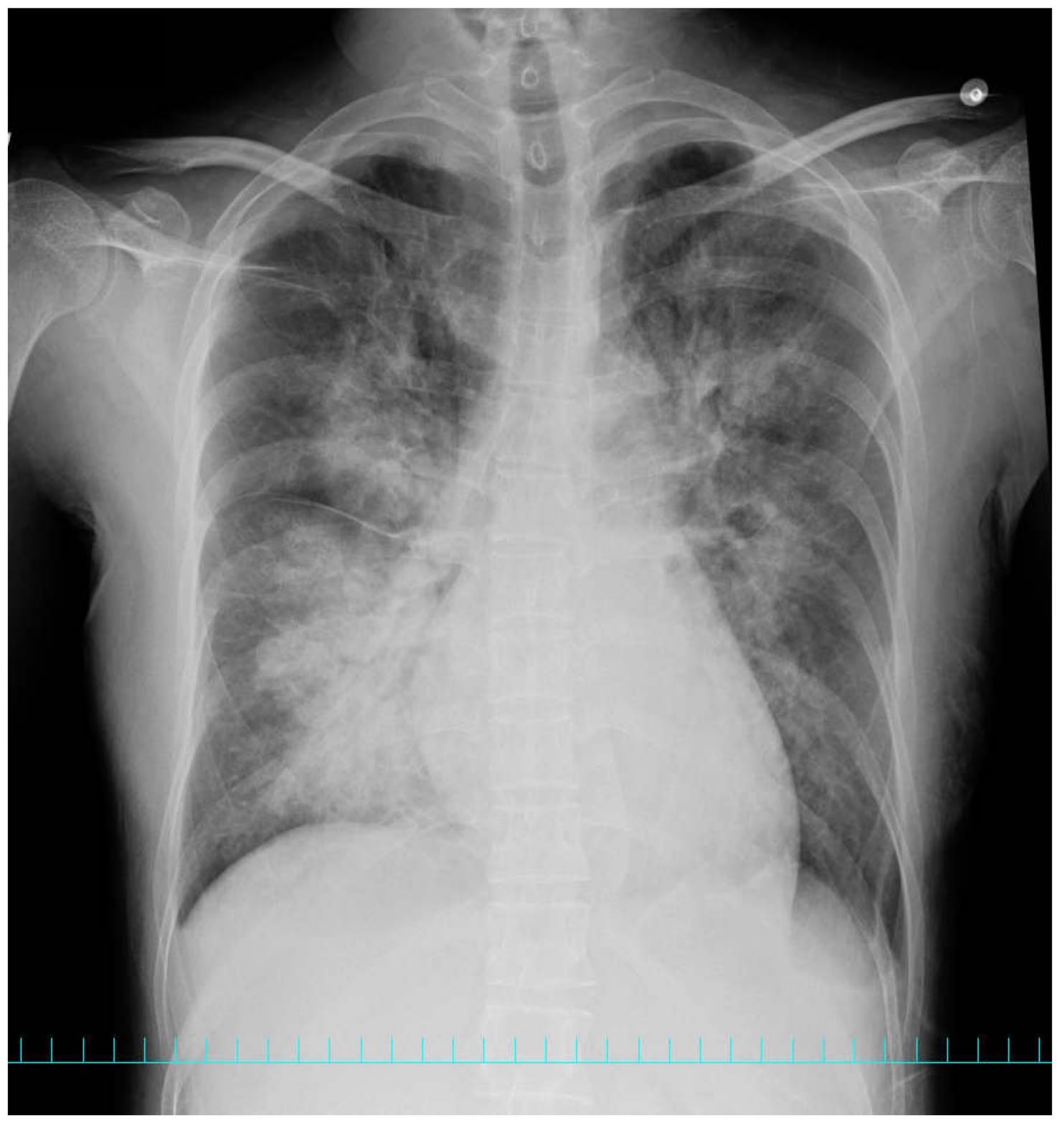
Chest X‐ray at the onset of heart failure: bilateral pulmonary infiltrates were observed.

Approximately 15 h postpartum, the patient developed respiratory distress with vital signs as follows: blood pressure, 130/80 mmHg; pulse, 130 bpm; and oxygen saturation, 82% on supplemental oxygen at 10 L/min. Additionally, chest X‐ray revealed pulmonary edema (Figure 2), echocardiography revealed severe cardiac dysfunction with a left ventricular ejection fraction of 15%, while coronary angiography revealed no significant stenosis, and an endomyocardial biopsy of the right ventricle revealed no specific findings. In response to hypovolemia caused by massive obstetric hemorrhage, approximately 2,000 mL of intravenous fluids was administered, in addition to blood transfusion; however, the urine output remained low, at only 100 mL/h, indicating oliguria. The results of blood tests conducted at the onset of heart failure are shown in Table 3.

**TABLE 3:** Laboratory data at the onset of heart failure.

Hematologic values			Blood chemical values			Arterial blood gas analysis under O^2^ 10 L/min		
White blood cell	33,310/μL	H	C reactive protein	2.91 mg/dL	H	pH	7.328	
Red blood cell	4.62 × 10^6^/μL	L	AST	25 U/L		PCO^2^	31 mmHg	L
Hemoglobin	14.0 g/dL	L	ALT	8 U/L		PO^2^	166.4 mmHg	H
Hematocrit	41.50%	L	LDH	482 U/L	H	HCO^3^	15.9 mmol/L	
Platelets	41.3 × 10^4^/μL		Creatinine	2.40 mg/dL	H	Base excess	−8.7 mmol/L	
			BUN	42.0 mg/dl	H	Lactate	4.1 mmol/L	H
**Coagulation tests**			eGFR	19.5 mL/min/1.73m^2^				
PT‐INR	0.9		Uric acid	11.9 mg/dL	H			
APTT	23	L	Na	136 mEq/L				
AT‐III	104%		K	5.2 mEq/l	H			
Fibrinogen	541 mg/dL	H	Cl	102 mEq/l				
FDP	21.6 μg/mL	H	BNP	400.6 pg/mL	H			
D‐dimer	15.7 μg/mL	H	CK	96 U/L				
			CK‐MB	32 U/L	H			

Abbreviations: ALT, alanine aminotransferase; APTT, activated partial thromboplastin time; AST, aspartate aminotransferase; AT‐III, antithrombin‐III; BNP, brain natriuretic peptide; BUN, blood urea nitrogen; CK, creatine kinase; CRP, C‐reactive protein; eGFR, estimated Glomerular filtration rate; FDP, fibrin/fibrinogen degradation products; LD, lactate dehydrogenase; PT‐INR, prothrombin time‐international normalized ratio.

## 3 Differential Diagnosis, Investigation, and Treatment

The differential diagnoses of postpartum respiratory failure include preeclampsia, pulmonary embolism, amniotic fluid embolism, transfusion‐associated circulatory overload (TACO), and peripartum cardiomyopathy (PPCM). The present case was carefully evaluated in consideration of these differential diagnoses.

Preeclampsia was excluded as the patient had no hypertension, thrombocytopenia, hypofibrinogenemia, suggesting endothelial dysfunction, or hepatic impairment. Pulmonary embolism was also excluded, as echocardiography revealed no evidence of right heart failure, and symptom onset occurred before the first ambulation after delivery, which is atypical for pulmonary embolism. Amniotic fluid embolism was also considered unlikely given the absence of organ dysfunction other than pre‐existing renal impairment, and only mild abnormalities in coagulation parameters.

Differentiation from TACO was difficult; however, PPCM was suspected based on the marked reduction in left ventricular ejection fraction. The patient was subsequently diagnosed with PPCM, after which she was transferred to the cardiology department and treated with carperitide, carvedilol, losartan potassium, and bromocriptine. Carperitide, carvedilol, and losartan potassium were administered as standard therapy for heart failure, whereas bromocriptine was administered because it has been reported to be effective for PPCM. The patient was discharged on postpartum day 16.

**FIGURE 3:**
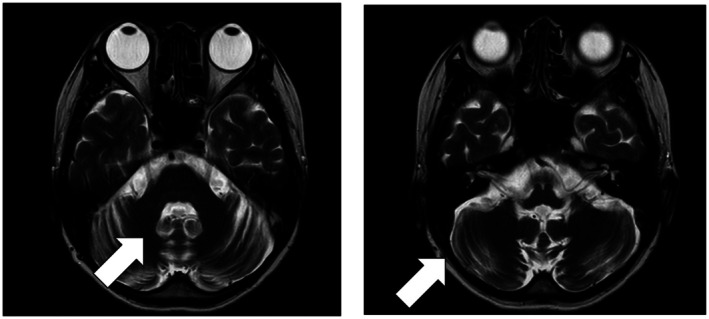
Head MRI showing atrophy of the brainstem and cerebellum.

Given the patient's history of diabetes mellitus, tubulointerstitial nephritis, preterm delivery, and cardiomyopathy, mitochondrial disease was suspected. Sensorineural hearing loss was detected by audiological testing, and magnetic resonance imaging of the brain revealed atrophy of the brainstem and cerebellum (Figure 3). A subsequent genetic analysis identified a m.3243A>G mutation, leading to a diagnosis of mitochondrial disease based on the diagnostic criteria provided by the Japanese Ministry of Health, Labour, and Welfare.

## 4 Conclusion and Results (Outcome and Follow‐Up)

The patient was started on taurine therapy to mitigate the mitochondrial disease symptoms, and is currently receiving treatment and appropriate follow‐up. Although uterine involution progressed favorably postpartum, a small retained placental fragment was noted within the uterine cavity. However, this remnant spontaneously resolved by postpartum day 45, at which point obstetric follow‐up was concluded. The patient was further counseled that a subsequent pregnancy was not recommended due to the potential risks associated with her underlying condition.

The neonate was admitted to the neonatal intensive care unit due to prematurity and low birth weight. Endotracheal intubation was required on day 0 due to respiratory distress syndrome; however, extubation was successfully conducted on day 1. Audiological testing revealed right‐sided hearing impairment, but cytomegalovirus polymerase chain reaction (PCR) testing was negative, and the patient is currently undergoing otolaryngological follow‐up. The infant was discharged home on day 81 of life in good condition.

## 5 Discussion

Mitochondrial diseases are disorders caused by abnormalities in the mitochondrial respiratory chain, leading to impaired adenosine triphosphate (ATP) production and affecting organs with high energy demands. These abnormalities are thought to be caused by mutations in mt‐ or nDNA. Recent epidemiological studies confirm that pathogenic mtDNA mutations are a major cause of human disease, affecting ≥ 1 in 5000 individuals [2, 3]. Mitochondrial diseases cause a variety of clinical symptoms across multiple organs, such as in the present case, and are classified based on clinical presentation into conditions such as mitochondrial myopathy, encephalopathy, lactic acidosis, and stroke‐like episodes (MELAS), myoclonic epilepsy with ragged‐red fibers (MERRF), maternal inherited diabetes‐deafness syndrome (MIDD), and mitochondrial nephropathy (Mit N). Several causative genetic mutations have been reported, with m.3243A>G being the most prevalent, observed in approximately 80% of MELAS cases [4, 5].

There is no unified consensus on the impact of mitochondrial disease on pregnancy; however, various insights have been gained from retrospective studies and case reports. Amel et al. conducted a retrospective study of 103 women diagnosed with mitochondrial disease, reporting miscarriage at a rate of 29.2%, new‐onset high blood pressure at 24.3%, gestational diabetes at 23.3%, small‐for‐gestational‐age fetuses at 21.4%, and preeclampsia at 18.4%. Additionally, symptoms such as fatigue, muscle pain, cramps, weakness, and nausea worsened during pregnancy [6]. Yanagawa reported a case of MERAS with deterioration during pregnancy and concluded that the metabolic changes during pregnancy increase the stress on mitochondrial function. They also suggested that metabolic changes during pregnancy can exacerbate mitochondrial dysfunction in mitochondrial disorders [7].

Feeney et al. analyzed 149 deliveries among 67 women diagnosed with mitochondrial disease and reported that the m.3243A>G mutation group had significantly higher rates of gestational diabetes, respiratory distress, and hypertension as well as an increased rate of preterm births, with 53.3% occurring before 37 weeks and 12.9% occurring before 32 weeks of gestation. They also mentioned that the normal physiological adaptations observed during pregnancy are likely to have bioenergetic consequences that increase the need for mitochondrial ATP production. In the context of mitochondrial insufficiency, these additional demands on the ATP supply during pregnancy may be a key mechanism in maternal and fetal complications [8]. Dessole et al. reported a case of emergency hysterectomy in a patient with mitochondrial myopathy, from which they concluded that mitochondrial dysfunction might be related to uterine atony [9].

In our case, there were no indications of other factors, such as chorioamnionitis or cervical insufficiency, to explain the preterm birth, suggesting that the patient's mitochondrial disease contributed to her preterm delivery. Mitochondrial diseases are characterized by abnormalities in calcium‐dependent metabolic mechanisms of the mitochondrial membrane; therefore, magnesium sulfate, which antagonizes calcium, is contraindicated in these cases [10]. During a previous pregnancy, the patient developed respiratory distress immediately after the administration of magnesium sulfate—which was subsequently discontinued—thought to be influenced by mitochondrial disease. In this case, tocolysis was achieved using ritodrine, and no adverse events were observed; however, postpartum heart failure occurred after a blood transfusion, raising the possibility of transfusion‐associated circulatory overload. Idiopathic peripartum cardiomyopathy was also considered; however, the exact cause could not be determined. A myocardial tissue biopsy did not reveal any specific changes, and a definitive diagnosis was not established. Bromocriptine, a drug inhibiting prolactin secretion, is a targeted therapy available for PPCM [11].

The combination of relatively rare conditions, such as preterm birth (before 30 weeks of gestation), placenta accreta, and cardiomyopathy, suggests the need to consider underlying systemic diseases that could explain these findings. In this case, we arrived at the possibility of mitochondrial disease; however, there have been no reports on the association between mitochondrial disease and placenta accreta. Our patient experienced repeated difficulty with placental detachment; although placenta accreta was clinically suspected due to firm adherence, histopathological examination in the present pregnancy did not confirm the diagnosis of accreta. Interestingly, this occurred despite the absence of any known risk factors, indicating a potential association with mitochondrial disease. Several mechanisms may have contributed to the abnormal placental separation. First, defective decidualization and impaired endometrial remodeling may have promoted abnormal adherence. Second, chronic kidney disease and diabetes mellitus may have led to endothelial dysfunction and microvascular abnormalities, further disturbing placental detachment. Third, repeated manual removal of the placenta in prior pregnancies may have resulted in uterine scarring, providing a mechanical basis for impaired separation. Moreover, while mitochondrial dysfunction may not directly cause placenta accreta, it could indirectly contribute by driving oxidative stress and abnormal vascular formation [12]. Taken together, we suggest these factors may have acted synergistically to impair normal placental separation in this patient; however, further studies are needed to clarify these multifactorial mechanisms.

The m.3243A>G mutation is known to cause mitochondrial dysfunction by inducing a taurine modification defect in the anticodon of mitochondrial transfer ribonucleic acid (tRNA), thereby impairing the translation of the complex proteins in the electron transport chain [13, 14, 15] While physical fatigue, excessive carbohydrate intake, and infections are known to exacerbate MELAS, pregnancy itself is also suspected to be a contributing factor to the worsening of the condition. Even in healthy women, lactate levels increase during pregnancy, leading to the speculation that metabolic changes associated with pregnancy may further impair mitochondrial function.

Several studies have demonstrated that high‐dose oral taurine supplementation is effective and safe for the prevention of stroke‐like episodes in patients with MELAS [16, 17], and since 2019, has been actively used in the treatment of patients with MELAS in Japan. Although the effects of taurine supplementation in pregnant women with mitochondrial disease remain unclear, it may improve the aforementioned pregnancy complications, warranting further investigation.

There are currently no established criteria for permitting pregnancy in women with mitochondrial disease. Therefore, clinical decision‐making should be individualized, with particular attention paid to the degree of organ involvement, such as cardiac dysfunction, renal impairment, or neurologic deficits, given that these factors strongly correlate with adverse obstetric and maternal outcomes [18]. In addition, when discussing contraception, combined oral contraceptives should be approached with caution in this population owing to their potential to exacerbate cardiovascular, thrombotic, or metabolic risks. Non‐estrogen methods, such as intrauterine devices, are generally considered safer alternatives in women with significant organ involvement or other risk factors.

Mitochondrial diseases exhibit a wide range of symptoms and severities, suggesting the possibility that a substantial number of mild cases go undiagnosed, especially as public awareness of these diseases is low and they are not commonly recognized by physicians. In our case, if the patient had been diagnosed with mitochondrial disease prior to becoming pregnant, preventive measures might have improved the outcomes of her pregnancy. It is crucial, therefore, to consider mitochondrial disease in the differential diagnosis of young individuals who present with abnormalities across multiple organs.

## Author Contributions

**Tomoyuki Watanabe:** conceptualization, writing – original draft. **Gen Ishikawa:** supervision. **Tsutomu Tabata:** supervision.

## Funding

No funds, grants, or other support were received to assist with the preparation of this manuscript.

## Ethics Statement

Ethics approval was not required for this case report in accordance with the policy of Tokyo Women's Medical University.

## Consent

Written informed consent was obtained from the patient for the publication of this case report.

## Conflicts of Interest

The authors declare no conflicts of interest.

## Data Availability

Data sharing not applicable ‐ no new data generated, or the article describes entirely theoretical research.

## References

[1] S. Parikh , A. Goldstein , M. K. Koenig , et al., “Diagnosis and Management of Mitochondrial Disease: A Consensus Statement From the Mitochondrial Medicine Society,” Genetics in Medicine 17, no. 9 (2015): 689–701. 25503498 10.1038/gim.2014.177 PMC5000852

[2] G. S. Gorman , A. M. Schaefer , Y. Ng , et al., “Prevalence of Nuclear and Mitochondrial DNA Mutations Related to Adult Mitochondrial Disease,” Annals of Neurology 77, no. 5 (2015): 753–759. 25652200 10.1002/ana.24362 PMC4737121

[3] L. M. Cree , D. C. Samuels , and P. F. Chinnery , “The Inheritance of Pathogenic Mitochondrial DNA Mutations,” Biochimica et Biophysica Acta 1792, no. 2 (2009): 1097–1102. 19303927 10.1016/j.bbadis.2009.03.002 PMC2785871

[4] Y. Goto , I. Nonaka , and S. Horai , “A Mutation in the tRNA(Leu)(UUR) Gene Associated With the MELAS Subgroup of Mitochondrial Encephalomyopathies,” Nature 348 (1990): 651–653. 2102678 10.1038/348651a0

[5] A. W. El‐Hattab , M. Almannai , and F. Scaglia , “MELAS,” in GeneReviews® [Internet], ed. M. P. Adam , S. Bick , G. M. Mirzaa , R. A. Pagon , S. E. Wallace , and A. Amemiya (University of Washington, Seattle, 2001), 1993–2025, https://www.ncbi.nlm.nih.gov/books/NBK1233/. 20301411

[6] A. Karaa , I. Elsharkawi , M. A. Clapp , and C. Balcells , “Effects of Mitochondrial Disease/Dysfunction on Pregnancy: A Retrospective Study,” Mitochondrion 46 (2019): 214–220. 29990538 10.1016/j.mito.2018.06.007

[7] T. Yanagawa , H. Sakaguchi , T. Nakao , et al., “Mitochondrial Myopathy, Encephalopathy, Lactic Acidosis, and Stroke‐Like Episodes With Deterioration During Pregnancy,” Internal Medicine 37, no. 9 (1998): 780–783. 9804089 10.2169/internalmedicine.37.780

[8] C. L. Feeney , A. Z. Lim , E. Fagan , et al., “A Case‐Comparison Study of Pregnant Women With Mitochondrial Disease ‐ What to Expect?,” BJOG: An International Journal of Obstetrics & Gynaecology 126, no. 11 (2019): 1380–1389. 30801962 10.1111/1471-0528.15667 PMC6767368

[9] S. Dessole , G. Capobianco , G. Ambrosini , and G. Battista Nardelli , “Postpartum Hemorrhage and Emergency Hysterectomy in a Patient With Mitochondrial Myopathy: A Case Report,” Archives of Gynecology and Obstetrics 267, no. 4 (2003): 247–249. 12592430 10.1007/s00404-002-0382-8

[10] K. Berkowitz , A. Monteagudo , F. Marks , et al., “Mitochondrial Myopathy and Preeclampsia Associated With Pregnancy,” American Journal of Obstetrics and Gynecology 161, no. 1 (1990): 146–147. 10.1016/0002-9378(90)90837-w 2301482

[11] G. Iannaccone , F. Graziani , P. Kacar , et al., “Diagnosis and Management of Peripartum Cardiomyopathy and Recurrence Risk,” International Journal of Cardiology Congenital Heart Disease 17 (2024): 100530. 39711771 10.1016/j.ijcchd.2024.100530 PMC11657248

[12] Y. Correia , J. Scheel , S. Gupta , and K. Wang , “Placental Mitochondrial Function as a Driver of Angiogenesis and Placental Dysfunction,” Biological Chemistry 402, no. 8 (2021): 887–909. 34218539 10.1515/hsz-2021-0121

[13] T. Suzuki , T. Suzuki , T. Wada , K. Saigo , and K. Watanabe , “Taurine as a Constituent of Mitochondrial tRNAs: New Insights Into the Functions of Taurine and Human Mitochondrial Diseases,” EMBO Journal 21, no. 23 (2002): 6581–6589. 12456664 10.1093/emboj/cdf656 PMC136959

[14] T. Yasukawa , T. Suzuki , N. Ishii , T. Ueda , S. Ohta , and K. Watanabe , “Defect in Modification at the Anticodon Wobble Nucleotide of Mitochondrial tRNA(Lys) With the MERRF Encephalomyopathy Pathogenic Mutation,” FEBS Letters 467 (2000): 175–178. 10675533 10.1016/s0014-5793(00)01145-5

[15] T. Yasukawa , T. Suzuki , N. Ishii , S. Ohta , and K. Watanabe , “Wobble Modification Defect in tRNA Disturbs Codon‐Anticodon Interaction in a Mitochondrial Disease,” EMBO Journal 20, no. 17 (2001): 4794–4802. 11532943 10.1093/emboj/20.17.4794 PMC125593

[16] M. Rikimaru , Y. Ohsawa , A. M. Wolf , et al., “Taurine Ameliorates Impaired the Mitochondrial Function and Prevents Stroke‐Like Episodes in Patients With MELAS,” Internal Medicine 51 (2012): 3351–3357. 23257519 10.2169/internalmedicine.51.7529

[17] Y. Ohsawa , H. Hagiwara , S. I. Nishimatsu , et al., “Taurine Supplementation for Prevention of Stroke‐Like Episodes in MELAS: A Multicentre, Open‐Label, 52‐Week Phase III Trial,” Journal of Neurology, Neurosurgery & Psychiatry 90, no. 5 (2019): 529–536. 29666206 10.1136/jnnp-2018-317964 PMC6581075

[18] J. Finsterer , “Obstetric Involvement in Mitochondrial Disorders: A Review,” Medicine (Baltimore) 102, no. 11 (2023): e33336. 36930069 10.1097/MD.0000000000033336 PMC10019216

